# Architecture of the subthalamic nucleus

**DOI:** 10.1038/s42003-023-05691-4

**Published:** 2024-01-10

**Authors:** Asheeta A. Prasad, Åsa Wallén-Mackenzie

**Affiliations:** 1 University of Sydney, School of Medical Sciences, Faculty of Medicine and Health, Sydney, NSW Australia; 2 Uppsala University, Department of Organism Biology, 756 32 Uppsala, Sweden

**Keywords:** Cellular neuroscience, Basal ganglia

## Abstract

The subthalamic nucleus (STN) is a major neuromodulation target for the alleviation of neurological and neuropsychiatric symptoms using deep brain stimulation (DBS). STN-DBS is today applied as treatment in Parkinson´s disease, dystonia, essential tremor, and obsessive-compulsive disorder (OCD). STN-DBS also shows promise as a treatment for refractory Tourette syndrome. However, the internal organization of the STN has remained elusive and challenges researchers and clinicians: How can this small brain structure engage in the multitude of functions that renders it a key hub for therapeutic intervention of a variety of brain disorders ranging from motor to affective to cognitive? Based on recent gene expression studies of the STN, a comprehensive view of the anatomical and cellular organization, including revelations of spatio-molecular heterogeneity, is now possible to outline. In this review, we focus attention to the neurobiological architecture of the STN with specific emphasis on molecular patterns discovered within this complex brain area. Studies from human, non-human primate, and rodent brains now reveal anatomically defined distribution of specific molecular markers. Together their spatial patterns indicate a heterogeneous molecular architecture within the STN. Considering the translational capacity of targeting the STN in severe brain disorders, the addition of molecular profiling of the STN will allow for advancement in precision of clinical STN-based interventions.

## Abstract (web summary)

The subthalamic nucleus is a highly evolutionary conserved brain region. Emerging studies reveal spatio-molecular heterogeneity leading to improved understanding of the organization of the STN and targeting for specific neurological disorders.

## Introduction

The subthalamic nucleus (STN) is part of the extended basal ganglia and is a highly evolutionary conserved brain area identified in lampreys, rodents, cats, and primates^1^. The STN was first described in humans by French neurologist Jules Bernard Luys in 1865; with early literature references to body of Luys or *corpus Luysii*^2^. Its importance in motor function was later identified by James Martin in 1927, where a patient with damaged STN displayed abnormal involuntary movement (hemichorea); also subsequent cases of hemichorea showed similar STN pathology^3^. Ipsilateral STN lesions in non-human primate led to contralateral dyskinesias of the upper and lower limbs^4^, whereas lesion of the STN in non-human primate model of Parkinson’s disease (PD) reversed the motor deficits^5^. Later, deep brain stimulation (DBS) in the STN was demonstrated as a successful approach to reduce motor symptoms in PD patients^6^. In some cases, such STN-DBS was superior to pharmacological therapy even in PD patients with early motor complications^7^. Currently, STN-DBS is also applied as treatment of additional neurological and neuropsychiatric disorders, including obsessive-compulsive disorder (OCD)^8–10^ and dystonia^11^. STN is also a promising new target to treat refractory Tourette’s syndrome^12–15^. In addition, STN has been proposed as a target area for the treatment for drug addiction^16–19^. The successful targeting of STN in both motor and non-motor disorders is evidence of the complex function of the STN.

Parallel to symptom alleviation, some PD patients living with STN-DBS have reported adverse side-effects, including impact on cognitive, physiological, and limbic functions^20^. Both male and female PD patients report depression and weight gain while other symptoms show sex-differences. For example, sexual dysfunction and aggression were mainly reported by male patients^21–25^. However, further analysis of clinical reports will be required to identify the origin of adverse symptoms and to allow a segregation between PD-related symptoms and DBS-induced effects.

In addition to STN, also globus pallidus (GP) is an important target area for DBS electrodes in PD and dystonia^26^. Comparative reports of dystonia cases found that targeting of either STN or GP interna (GPi) is effective for dystonia. However, STN-DBS was superior for generalized dystonia and GPi-DBS was effective for truncal dystonia^27,28^. A comparison of adverse impact between brain regions indicated that targeting the STN relatively worsened the condition of apathy compared to GPi-DBS^29^. Retrospective studies comparing DBS in STN and GPi in PD show improvement on short-term motor symptoms, yet among STN-DBS treated patients there were incidence of suicide and impact on mood and quality of life^30,31^.

Due to established surgical protocols and successful outcomes improving debilitating neurological disorders, the STN is already a clinically important brain region in relation to motor, limbic and cognitive functions. In order to further refine treatments targeting the STN, better understanding of STN anatomy and its cellular and molecular composition should be of essence. For example, enhanced specificity and precision can be achieved by allowing such organizational knowledge improve the level of detail in stereotaxic brain maps implemented for surgical procedures in the STN. Further, any methodological developments towards cell-specific targeting of the STN might have the potential to reduce adverse side-effects and/or impact on non-motor symptoms.

### Anatomy

Anatomically the STN is described as a compact nucleus located between the thalamus and midbrain, ventral to the zona incerta (ZI) and adjacent to the substantia nigra (SN), in close vicinity of fields of Forel H^3^. Its signature biconvex lens shape is similar across human, rat, cat, and monkey^3,32^. The STN has an oblique orientation with three anatomical axes that run along the rostrocaudal, mediolateral and dorsoventral axis^33,34^.

On average, there are 225,000 neurons per hemisphere in the human STN^35–37^. Magnetic resonance imaging (MRI) approaches report that the volume of the human STN is mainly between 50–80 mm^3^ with no difference between hemispheres or decrease with aging^38,39^. However other MRI techniques show a larger volume of ~155 mm^3^, within the range of 120 mm^3^ to 175 mm^3,^^40^. The variation in range could be contributed by different magnetic resonance imaging (MRI) techniques such as ultra-high field 7 tesla (T) and o3T MRI^41^. Moreover, the STN comprises a relatively small brain region and its boundaries are surrounded by dense fibres of the internal capsule, ansa lenticularis and lenticular fasciculus^42^.

MRI techniques show compartmentalization of the STN anatomical regions^40^. These have been referred to as motor, associative and limbic regions, forming the so called tripartite model of STN, correlating anatomy with function^43,44^. These STN compartments are also known in the literature as territories, domains, subdivisions, subareas. The STN compartments run through the rostrocaudal, mediolateral and dorsoventral axes^42^. The dorsolateral compartment is associated with sensorimotor function, the ventromedial (or central) compartment with cognitive/associative function, and the medial-most compartment with limbic function^45^. The limbic area of the STN is often referred to as the limbic tip, and it is directly associated with the para-STN towards which no specific anatomical border is apparent.

The anatomical subdivisions within the STN structure substantially overlap between functions^46^. It has been observed that, in monkeys, these compartments, particularly the associative and limbic, do not have well-defined boundaries, but are separated by functional gradients^47^. Emerging molecular marker and MRI studies in the STN challenge the tripartite model and the presence of three subdivisions of the STN^44,48,49^. A detailed analysis of publications from primate studies between 1925 to 2010 identified everything from zero to four subdivisions within the STN, and reported limited evidence to support the three subdivisions^49^. Further, in a recent report, six subparts were arbitrarily outlined (in cresyl violet stained tissue sections, i.e. not based on molecular markers); No 1 for the anteromedial part, No. 2 for the central part (further subdivided into centrodorsal (2d) and centroventral (2v), No 3 for lateral part (further subdivided into laterodorsal (3d) and lateroventral (3 v) and No 4 for posterolateral part^50^. Evidently, the internal organization of the STN is still under intense investigation, and more studies addressing the cellular, anatomical, structural organization from different aspects are likely to appear in literature in the near future.

A number of expression studies performed in human STN this millennium has identified new markers, rostro-caudal gradient expressions, and differences in neural densities along dorsal and ventral regions^33,34,51,52^. Together the rapidly expanding literature on STN organization supports further subdivisions within the STN which are defined by a combination of anatomy and spatial expression of molecular and/or cellular markers. The understanding of the internal organization of the STN is highly valuable towards clinical targeting of the STN, not least for refining treatment approaches. This includes the DBS technique which is dependent on anatomical specification to enable optimized implantation sites for electrode stimulation. Merging knowledge gained in recent studies may be an important step to develop an inclusive organization of the STN, including a common terminology to describe this internal organization.

**Fig. 1:**
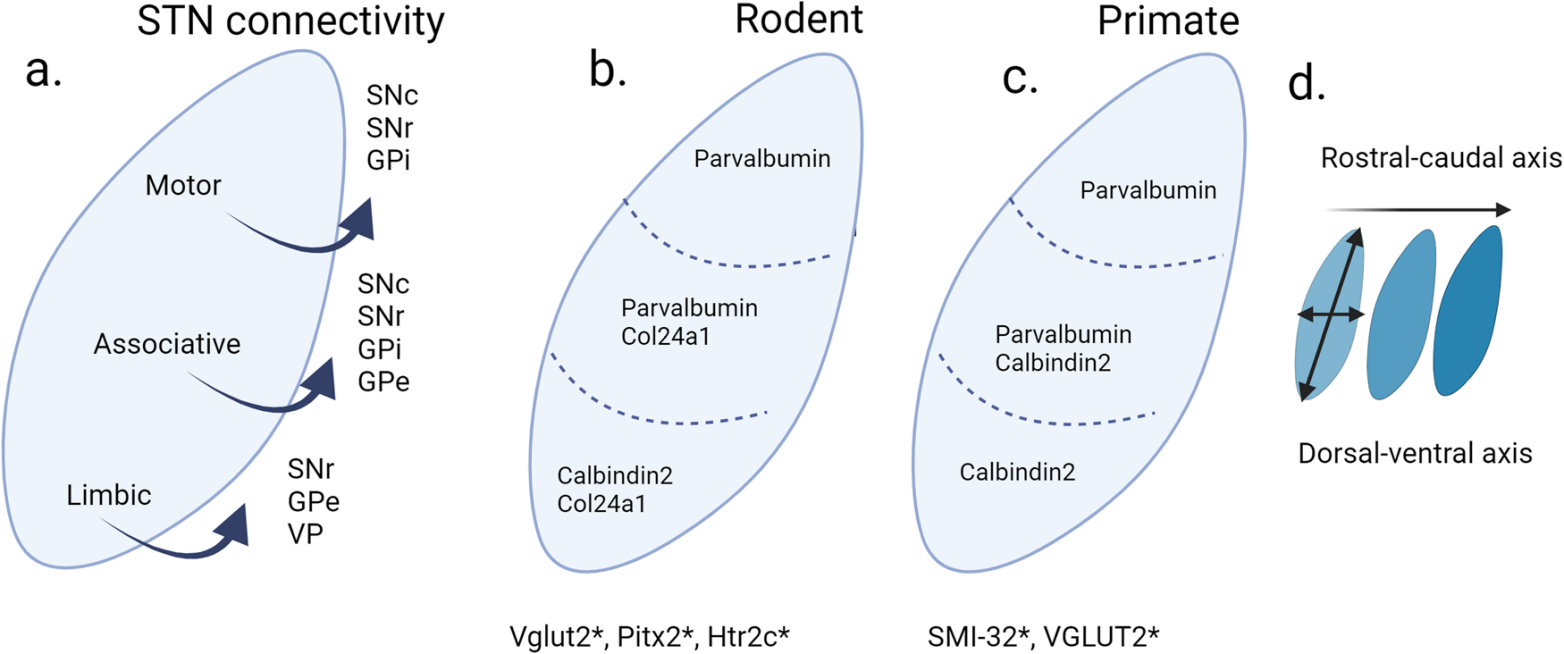
The subthalamic nucleus (STN) outlined with connectivity and selected gene expression. **a** Anatomical subdivisions are topologically organized with STN efferents based on published neural tracing studies by Carpenter et al., 1968, Parent et al., 1989, Nambu et al., 1996 and Karachi et al., 2004. **b** Selected gene expression (Parvalbumin, Calbindin 2, Col24a1: detected as mRNA or protein) in mouse (**c**) and primate based on Hardmann et al., 1997 (**d**). expression patterns are altered across rostral-caudal and the dorsal-ventral axis * indicates global STN gene expression. For details and full nomenclature, see the main text. Image created in Biorender.

Neural tracing and MRI studies of human and non-human primates confirm that the three proposed STN subdivisions have topologically organized connectivity to the basal ganglia (striatum and GP) and extended regions including substantia nigra (SN), pedunculopontine nucleus and ventral pallidum (VP)^40,53^ (Fig. 1A). In addition, the use of MRI has proven effective in demonstrating that motor network connectivity is predictive of effective subthalamic stimulation^54,55^. Human STN receives input from the orbitofrontal, prefrontal and limbic cortices as well as the motor cortex^43,55–57^.

In vivo diffusion weighted MRI technology to analyze the white matter connectivity of the STN from 12 healthy adults showed three distinct subregions defined by connectivity to cortical and subcortical regions^40^. In addition to the expected major dense cortical projections, overlapping networks between the motor and limbic connectivity were also identified^40,46^. Applying a similar structural connectivity-based parcellation protocol in 17 PD patients scheduled for STN-DBS surgery also showed three parcellations of the posterolateral motor region; and the limbic region located in the anteromedial part of the STN^58^. The convergence between these pathways may explain the impact on non-motor behaviors in PD patients treated with STN-DBS, and the importance of electrode placement within the STN for optimal motor improvements^59^.

PD patients are affected by several different non-motor symptoms that occur concurrently, or even precede, motor symptoms^60^. In addition to improving motor symptoms, DBS has been shown to be effective in relieving non-motor symptoms^20,61–63^. Further, improvement for some of these, including mood, apathy, attention, memory and sleep, has been shown to depend on the location of stimulation^64^.

On the other hand, adverse non-motor symptoms as side-effects are often reported from STN-DBS clinical patients^25,59,65^. For example, STN-DBS may induce hypomania, particularly when selected electrode contacts are located in the anteromedial part of the nucleus^66^, which is the region used with success to treat severe and resistant OCD^8^. These observations highlight the need for further understanding of the anatomical features of STN, and how this nucleus connects within the brain. Apart from the complex anatomy of STN, a striking heterogeneity in its transcriptomic landscape was recently revealed by spatio-molecular data originated from single-nuclei RNA-sequencing (snRNASeq) in mice^67^.

Various types of molecular, cellular, and anatomical heterogeneity, as well as connectivity, may be important to consider in the context of the beneficial effects of STN-DBS and also of adverse side-effects experienced as a consequence of this treatment. Alternative to anatomical targeting within the STN, cell-specific targeting is a futuristic approach^26,68^. Further, with rapid developments in neuromodulation technologies together with spatio-molecular mapping of STN, the potential of symptom-specific targeting with limited adverse impact is possible to foresee in the near future.

Overall, the STN shows a conserved anatomy across species with delineation of subregions which were initially proposed by functional domains^1,69^. However, the anatomical-functional organization of the STN remains to fully established. Anatomical tracing and MRI studies challenge the tripartite model. Below, we summarize cellular and molecular markers of the STN in primates and rodents, including neurotransmitter-related molecules and calcium-binding proteins as well as other, more recently described molecular markers within the STN. Thereafter, we address studies in which motor, associative and limbic functions of the STN have been addressed, followed by studies of the STN in neurological and neuropsychiatric disorders, including cellular changes and alterations in firing patterns.

### Cellular markers of the STN

The STN is a major excitatory brain nucleus^70^. STN neurons primarily use glutamate as neurotransmitter. Beyond the glutamatergic identity, immuno-histological studies show intermingled neurotransmitter receptor identities of GABAergic, serotonergic, and dopamine receptors, indicative of complex afferent neurotransmission reaching the STN^32,53,71–79^. The fundamental cellular composition of the STN comes from gene expression analysis derived from mRNA and protein localization within the structure. The summary presented here includes studies from rodent, non-human primates and humans (data summarized below is also listed in Table 1).

**Table 1:** Molecular marker expression in STN.

Molecular marker (gene expression detected as mRNA or protein)	Abbreviation (mRNA in italics (*mouse only*), protein in capitals)	Species analyzed	Site of location (STN unless otherwise stated) (mRNA or protein)	Reference
Adenylate cyclase activating polypeptide 1	*Adcyap1*	Mouse	Central STN	Wallén-Mackenzie et al. 2020^67^
A2A adenosine receptor	ADORA2A	Human	Distributed in decreasing dorsal to ventral gradient	Emmi et al., 2022^97^
BAI1 associated protein 3	*Baiap3*	Mouse	pSTN and surrounding hypothalamic structures	Wallén-Mackenzie et al., 2020^67^
Calcium voltage-gated channel auxiliary subunit alpha2 delta 3	*Cacna2d3*	Mouse	Below detection or absent	Wallén-Mackenzie et al., 2020^67^
Calbindin 1	CALB1	Human	Labelled on fibres Faint detection	Augood et al., 1999^73^ Munkle et al., 2000
Calbindin 2 also known as Calretinin	CALB2	Human	Intense immunoreactivity in ventral STN; both cell bodies and fibres. Highest detection in caudal aspect of STN.	Augood et al., 1999^73^; Munkle et al., 2000; Wu et al., 2017; Alkemade et al., 2019^33^, Bokulic et al., 2021
Calbindin 2	CALB2	Monkey	Both cell bodies and fibres	Fortin and Parent 1996^102^
Calbindin 2	*Calb2*	Mouse	Moderate in medial STN (STNa domain)	Wallén-Mackenzie et al., 2020^67^
Collagen type XXIV alpha 1 chain	*Col24a1*	Mouse	Strong medal detection (STNa), sparser in central STN (STNb)	Wallén-Mackenzie et al., 2020^67^
Dopamine receptor 1 and 2	D1R and D2R	Monkey	Presynaptic axons	Galvan et al., 2014^98^
Dopamine receptor 2	D2R	Human	Distributed in decreasing gradient along ventromedial-to-dorsal axis	Hurd et al., 2001^76^; Emmi et al., 2022^97^
Ferritin	FERR	Human	Distributed in oligodendrocytes within STN	Alkemade et al., 2019^33^
Fibroblast growth factor 11	*Fgf11*	Mouse	Throughout STN, low intensity	Wallén-Mackenzie et al., 2020^67^
Forkhead box protein P2	FOXP2	Human	Variable pattern within STN	Bokulic et al., 2021
GABA-A receptor subunits	GABA-A (a1, a3, b2/3, and g2)	Human	STN principal neurons	Wu et al., 2017
GABA-B receptor subunit	GABAB (R1 and R2)	Human	STN principal neurons	Wu et al., 2017
GABA-A receptor subunit alpha 3	GABRA3	Human	Mainly neuronal staining and punctate fibre staining	Alkemade et al., 2019^33^
GABA-B receptor 1	GABAB R1	Monkey	Moderate distribution throughout the rostrocaudal extent of the STN	Charara et al., 2000
GABA transporter 1	GAT1	Human	STN	Augood et al., 1999^73^
Glutamate decarboxylase 65/67	GAD65/67	Human	Parse positive neuronal staining, moderate fibre terminal staining (presynaptic boutons) in dorsolateral tip of STN	Wu et al., 2017; Alkemade et al., 2019^33^
Glutamate decarboxylase 1	*Gad1*	Mouse	Dorsal STN (STNds) and para-STN	Wallén-Mackenzie et al., 2020^67^
Glycine receptor alpha 3	*Glra3*	Mouse	Para-STN	Wallén-Mackenzie et al., 2020^67^
Glutamate receptor 1 and 2/3	GluR1 GluR2/3	Monkey	Cell bodies and synaptic clefts	Wang et al., 2000
Metabotropic glutamate receptor subtype 1 and 5	mGluR1a mGluR5	Monkey Rat	Strong labelling in neuropil and in postsynaptic STN neurons.	Kuwajima et al., 2004^53^ Awad et al., 2000^86^
N-methyl-D-aspartate receptor 1	NMDAR1	Monkey	Cell bodies and synaptic clefts	Wang et al., 2000
Neuromedin b receptor	*Nmbr*	Mouse	Throughout STN. Low detection.	Wallén-Mackenzie et al., 2020^67^
Neurexophilin 1	*Nxph1*	Mouse	Throughout STN	Wallén-Mackenzie et al., 2020^67^
Neurexophilin 4	*Nxph4*	Mouse	Throughout STN except for dorsal strip (STNds)	Wallén-Mackenzie et al., 2020^67^
Neurofilament H	SMI-32	Human	Uniformly detected	Wu et al., 2017; Alkemade et al., 2019^33^
Neuronal nitric oxide synthase	nNOS	Human	Most abundant neuronal marker in STN	Bokulic et al., 2021
NK2 homeobox 1	NKX2.1	Human	Ventral regions in rostral and caudal plane. Uniform in central STN.	Bokulic et al., 2021
Paired like Homeodomain 2 also known as pituitary homeobox 2	*Pitx2*	Mouse	Throughout STN	Martin et al., 2004^107^ Skidmore et al., 2008^109^ Schweizer et al., 2014^108^ Schweizer et al., 2016; Wallén-Mackenzie et al., 2020^67^; Serra et al., 2023^157^
Parvalbumin	PARV	Human	STN, primarily dorsolaterally/caudally.	Parent et al.,1996; Augood et al.,1999^73^; Munkle et al., 2000; Alkemade et al., 2019^33^, Bokulic et al., 2021
Parvalbumin	PV	Mouse	STN, dorsal and central (STNb,c domains). Note: In contrast to most other neurons, *PV*+ in STN neurons is mostly glutamatergic, (PV/Vglut2 colocalisation)	Wallén-Mackenzie et al., 2020^67^; Serra et al., 2023^157^
Paired box protein 6	PAX6	Human	Variable pattern	Bokulic et al., 2021
Potassium voltage-gated channel subfamily A regulatory beta subunit 3	*Kcnab3*	Mouse	Throughout STN. Co-localised with *Pitx2*	Wallén-Mackenzie et al., 2020^67^.
Serotonin	Serotonin / 5HT	Monkey	Highest density in medial and ventral STN	Mori et al.,1985^32^
Serotonin receptor subtype 2c	*Htr2c*	Mouse	Throughout STN; similar as Pitx2 and Vglut2	Wallén-Mackenzie et al., 2020^67^
Serotonin transporter	SERT	Human	Passing fibres, more staining in anterior STN as compared to posterior STN	Parent et al., 2011, Alkemade et al., 2019^33^
Syntaxin binding protein 2	*Stxbp2*	Mouse	Moderate in STN	Wallén-Mackenzie et al., 2020^67^
Synaptophysin	SYN	Human	Punctate staining throughout	Alkemade et al., 2019^33^
Synaptoporin	*Synpr*	Mouse	Below detection or absent	Wallén-Mackenzie et al., 2020^67^
Tachykinin precursor 1 (encoded four products, including Substance P)	*Tac1*	Mouse Monkey	Para-STN	Wallén-Mackenzie et al., 2020^67^; Serra et al., 2023^157^
Transferrin	TRANSF	Human	Oligodendrocytes and blood vessels in STN	Alkemade et al., 2019^33^
Tyrosine hydroxylase	TH	Human	None reported, passing fibres only	Hedreen 1999^74^; Alkemade et al., 2019^33^
Vesicular glutamate transporter 1	VGLUT1	Human	Dense at STN borders and punctate in fibres	Alkemade et al., 2019^33^
Vesicular glutamate transporter 2	VGLUT2	Monkey	Throughout STN	Santin et al., 2023^50^; Serra et al., 2023^157^
Vesicular glutamate transporter 2	*Vglut2*	Mouse	Throughout STN, co-expressed with *Pitx2*	Wallén-Mackenzie et al., 2020^67^; Schweizer et al., 2014^108^, Serra et al., 2023^157^

^*^Italics denotes mRNA detection, mouse. #Terminology STN domains (STNa, STNb, STNc, STNds), see Fig. 2 (originally described in Wallén-Mackenzie et al. ^67^)

***List of molecular markers in the STN***. Gene expression detected as mRNA or protein and reported in human, monkey, rodent STN. For details, see main text.

### Neurotransmitters

#### Efferent signaling from STN

*Glutamate*: Glutamatergic neurons are by far the most abundant cell type throughout the STN structure across species. In humans, the highest density of neurons positive for glutamatergic markers has been reported in the ventromedial part of the STN, with only a small population of GABAergic interneurons^80^. In mice, mRNA encoding the Vesicular glutamate transporter 2 (VGLUT2) is distributed throughout the STN^67,81^). A similar expression pattern of VGLUT2 mRNA was recently reported in macaque STN^50^.

Vesicular glutamate transporters (VGLUT1-3; encoded by the Slc17A7, Slc17A6, Slc17A8 genes, respectively) import glutamate into presynaptic vesicles and are the key cellular markers of glutamatergic excitatory neurotransmission. Since glutamate is an amino acid present in all cells, the presence of a VGLUT protein effectively means that the neuron in question can package and release glutamate from presynaptic vesicles upon depolarization and thus use its glutamate as neurotransmitter. Both VGLUT1 and VGLUT2 proteins have been identified as glutamate transporters in the membrane of pre-synaptic vesicles and are therefore considered as molecular markers defining the glutamatergic phenotype of neurons^82–84^. In terms of pre-synaptic vesicular transporters in neurons, distribution patterns of mRNA and protein will be strikingly different. While mRNA will be representing the cell soma of the encoding cell (and can be detected by in situ hybridization), the protein will be transported to the presynaptic terminal, and hence detected (by immunohistochemistry) at the synapse. Thus, in the mature neuron, the VGLUT protein will be found in a different brain area (in the pre-synpatic terminals synapsing on the target) than its corresponding mRNA (in the cell soma). This is particularly important to bear in mind when interpreting expression analysis of any vesicular transporter, including VGLUTs. Since VGLUT protein represents glutamatergic terminals, the presence of VGLUT protein in a structure thereby identifies incoming projections. Considering the STN of rodents and primates, the glutamatergic phenotype is molecularly identified by the presence of Vglut2 mRNA in the somata of STN neurons, while the corresponding VGLUT2 protein is found in their axonal terminals in target areas, including GP, VP, SN and pedunculopontine nucleus, as outlined above.

#### Afferent signaling to STN

##### Glutamate

In terms of glutamatergic afferent projections reaching the STN, analysis of human brain tissue has identified VGLUT1 protein, indicative of afferent terminals from the cortex (and possibly other excitatory brain areas positive for this subtype of VGLUT), localized to the dorso-caudal extent of the STN with punctate labelling of the fibers^33^. In monkey STN, AMPA receptor subunits GluR1, GluR2/3 and N-methyl-D-aspartate receptor 1 (NMDAR1) were detected^85^. Metabotropic glutamate receptor subtype 1 and 5 are located on STN neurons in both monkey and rats^53,86^.

##### GABA

GABAergic neurons, in STN considered interneurons^70^, have been reported to be significantly more numerous in the anteromedial STN than the rest of the STN structure in humans^36,80^. Glutamate decarboxylase (GAD65/67), a GABA marker, is sparsely detected on STN neurons with moderate fiber terminal staining (presynaptic boutons) in the dorsolateral tip of STN^33^. GABA_A_ (a1, a3, b2/3, and g2) and GABA_B_ (R1 and R2) receptor subunits are present on STN principal neurons. These GABA_A_ receptor subunits are detected in a gradient along the dorsolateral to ventromedial axis in humans^79^. Further, GABA receptor a3 subunits (GABRA3) are localized in the anterior ventromedial region of the STN^33^. Anterograde tracing experiments in non-human primates with immunohistochemistry reveal major GABA afferents from GP and VP^87–92^.

##### Serotonin

Serotonin transporter (SERT) is present in passing fibers in rat, cat, monkey and human STN. In monkeys and humans, SERT was abundantly detected in medial parts of the STN^32,33,50^. In humans, 5’HT-immunoreactive fibers display a decreasing gradient along the mediolateral axis^93^. In rats, autoradiography studies show that SERT and serotonin receptor subtypes 5´HT1B and 5´HT2C are present in the STN. Notably, 5´HT1A and 5´HT2A receptors were not detected^94^. In mice, mRNA for 5´HT2C receptor was reported to be present along the entire STN structure, in a pattern similar to Vglut2 mRNA^67^.

##### Dopamine

The STN receives sparse dopaminergic projections from the substantia nigra *pars compacta* (SNc) and ventral tegmental area (VTA) as reported in monkeys^95,96^ and humans^73^. Dopamine receptor subtype 2 (D2R) is distributed in the human STN in decreasing gradient along a ventromedial-to-dorsal axis^76,97^. D2R is co-localised with adenosine receptor A2A within the dorsal and medial regions of the human STN^97^. In non-human primates, Dopamine receptor subtype 1 (D1R) and D2R are distributed on presynaptic axons and putative glutamatergic and GABAergic terminals^98^. In rats, D1R has high expression^99^. Further, firing rates of STN neurons are not altered by D2R stimulation, yet increased by selective stimulation of DRD1^100^.

In addition to different neurotransmitter receptors localized on STN neurons, also other types of molecular markers defining different cellular functions have been demonstrated in the STN, reviewed below.

### Calcium-binding proteins

Calcium-binding proteins, specifically Parvalbumin (here abbreviated PARV), Calbindin (aka CALB1) and Calretinin (aka CALB2, Calbindin 2) are some of the major molecular markers examined in the STN in humans^36,52,73,79^. The distribution of these markers shows visible segmentation of PARV and CALB2 distribution in humans with approximately 50% of STN neurons positive for PARV and 50% CALB2-positive neurons^101^. These studies of the human STN also show that PARV is localized in the dorsal STN region and CALB2 in the ventral region of the STN, with some overlap or centrally^33,36,52,73^. This contrasting pattern of PARV and CALB2 has also been reported in monkeys. Similar to protein localization, their detection at the mRNA level shows similar distribution in the human STN^73,102^. This distinguishing molecular partition leads into three molecularly defined subdivisions (Parvalbumin^+^; Parvalbumin^+^/Calbindin2^+^; Calbindin2^+^) with distinctive anatomical localization (lateral-most; central; medial-most)^36,47,103–105^ (Fig. 1B–D).

Some functional features were supportive of the tripartite model of STN, in which the three anatomical subdivisions correlate with the three major STN functions; sensorimotor, associative and limbic^44^ (Fig. 3A). It has been proposed that PARV-positive STN neurons process motor functions whereas the CALB2-positive neurons are involved in limbic function. Moreover, this segmented localization of calcium-binding proteins suggests that differential calcium dynamics occurs along the STN^73^.

Summarizing, the STN is an excitatory nucleus, with complex afferent interactions with other neurotransmitter systems. Moreover, the spatial distribution of calcium-binding proteins indicates differential functional capacity across the STN. Together, expression of neurotransmitter markers, including cognate receptors and calcium-binding proteins, reveals complex regulation of neural transmission within the STN.

### Novel gene expressions detected within the STN of rodents

Similar to the human STN, the rodent STN shares the biconvex morphology and distribution of some cellular markers, including the distribution of Parvalbumin localized at the dorsal aspect and Calbindin 2 towards the ventral aspect of the STN^33,36,52,67,73^. However, there are also striking differences between rodent and primate, discussed further below.

The advantage of rodent studies is the availability of transgenics technology to label and manipulate specific genes and cells of interest within defined anatomical regions. Recent studies using fluorescent in situ hybridization (FISH) combining probes selective for several different markers show that *Pitx2* mRNA, encoding the transcription factor Paired Like Homeodomain 2 (PITX2), is neatly localized within the STN and has overlapping expression with *Vglut2* mRNA^81^. In fact, in the mouse STN, *Pitx2* and *Vglut2* mRNAs show 100% overlap^106^. Complete knock-out of the *Pitx*2 gene (Pitx2^−/−^) during embryonic development leads to abnormal development of the STN^107^. Two subsequent studies described the conditional knockout of *Vglut2* in all *Pitx2*-positive STN neurons^81,108^. The implementation of Cre-driven mouse genetics achieved by crossing transgenic Pitx2-Cre recombinase mice^109^ with targeting mice carrying a floxed *Vglut2* gene^110^, led to an overall reduction of 40% of *Vglut2* expression levels in the STN^108^. Functionally, this conditional blunting of glutamatergic neurotransmission from the STN resulted in decreased post-synaptic currents, behavioral hyperlocomotion (further discussed in^111^) and decrease in sugar consumption with a significant effect on the midbrain dopamine system. These knockout studies thereby demonstrated opposite roles of STN glutamatergic neurons in motor compared to motivated behavior^81,108^.

**Fig. 2:**
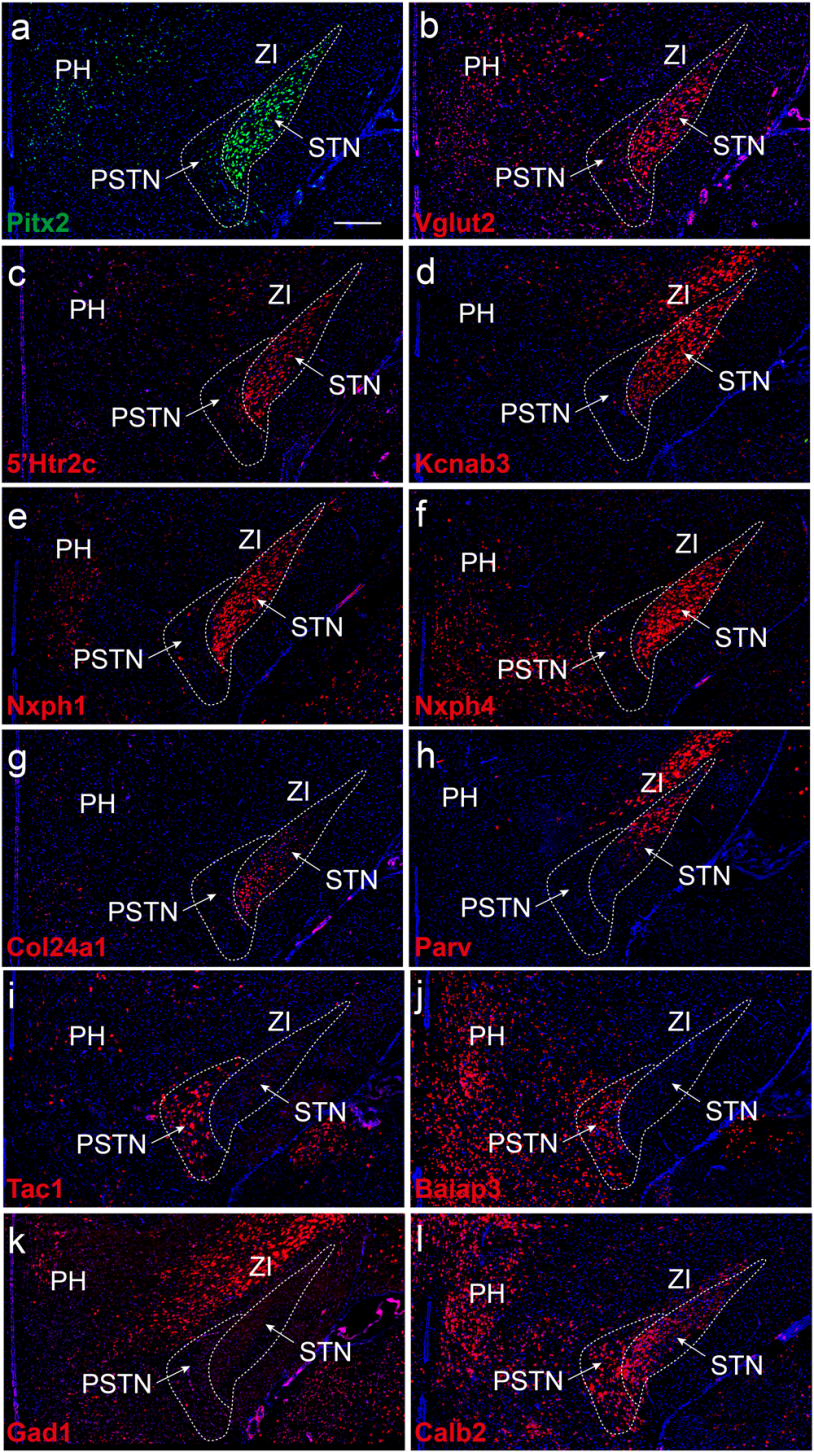
Molecular heterogeneity of mouse STN as revealed by single nucleotide transcriptomics followed through with spatial mRNA mapping. Serial mouse brain sections analyzed by fluorescent in situ hybridization (FISH) detects differential patterns and profiles of selected gene expression products; distribution within, and immediately surrounding, the STN structure: **a** Pitx2; **b** Vglut2; **c** Serotonin receptor subtype 2c, 5´Htr2c; **d** Kcnab3; **e** Nxph1; **f** Nxph4; **g** Col24a1; **h** PV **i** Tac1; **j** Baiap3; **k** Gad1; **l** Calb2. Note: Pitx2, Vglut2, Htr2c, Kcnab3, Nxph1 mRNAs (**a**–**e**) are present throughout the entire STN structure; Nxph4 mRNA is similar but excluded from dorsolateral-most part of STN; Col24a1 primarily in medially and centrally located STN neurons and excluded dorsally with opposite pattern of PV mRNA, primarily in dorsally and centrally located STN neurons while excluded medially (compare **g** and **h**); Tac1 and Baiap3 mRNAs absent from STN (**i** and **j**), but Tac1 strong in para-STN (PSTN), Baiap3 in PSTN and also surrounding hypothalamus (lateral and posterior hypothalamus, PH, LH); Gad1 (**k**; GABA marker) largely absent from STN and PSTN (in accordance with their excitatory phenotype) but present in GABAergic structure zona incerta (ZI), Calb2 mRNA stronger in medial STN and para-STN (PSTN) than dorsal STN (**l**). For mRNA full name, see the main text. See original publication for details (Wallén-Mackenzie et al. ^67^; published under Open access).

**Fig. 3:**
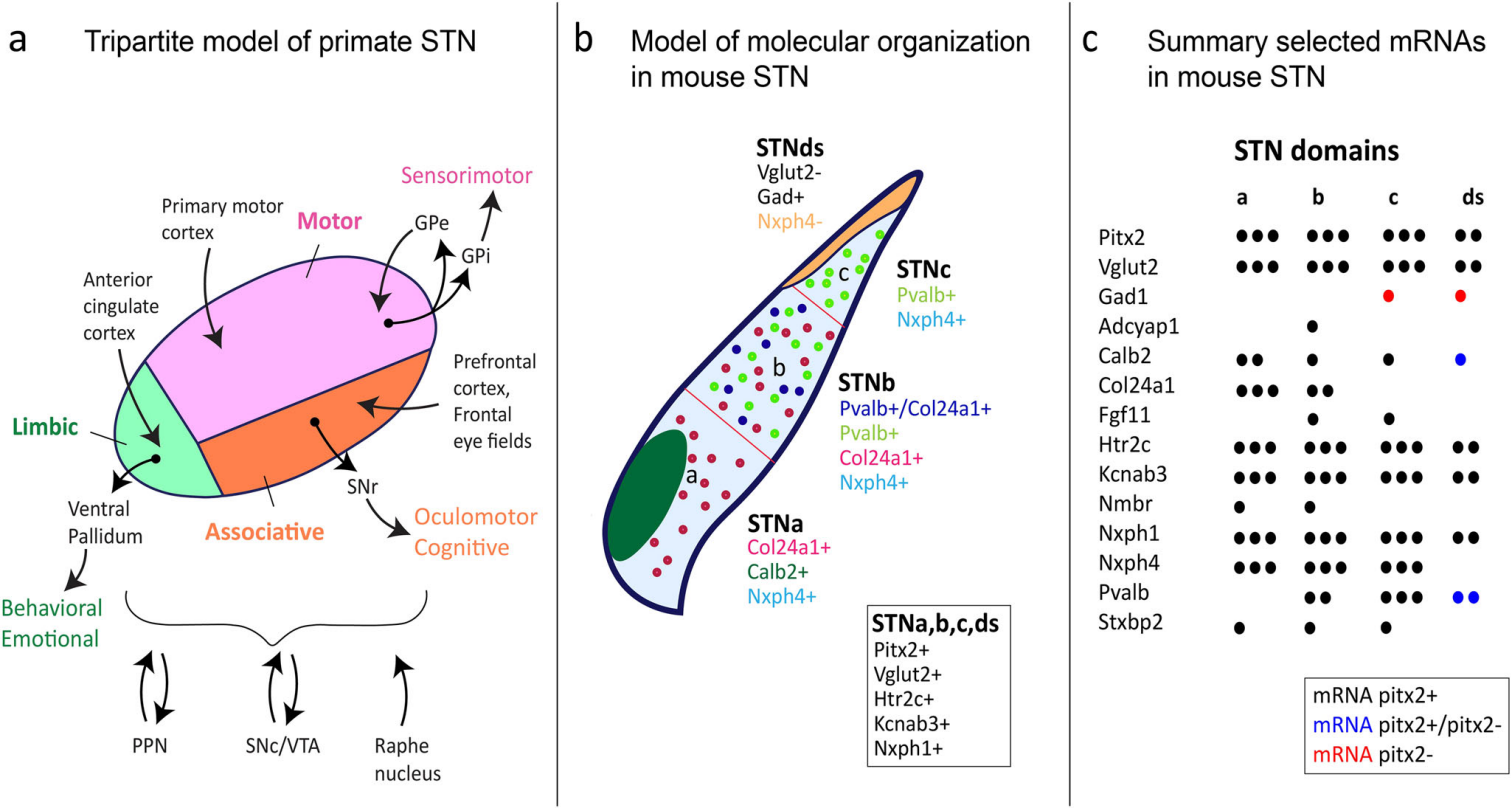
Tripartite model of primate STN presented next to a recent model of molecular organization within mouse STN. **a** Tripartite model of primate STN; motor, associative and limbic territories based on multiple anatomical-functional studies (references in main text). Drawing based on original illustration in Benarrouch, 2008; here updated with information of afferent and efferent projections as outlined by Baunez et al., 2016. Abbreviations: GPe, globus pallidus externa; GPi, globus pallidus interna; SNr, substantia nigra pars reticulata; SNc, substantia nigra pars compacta; PPN, pedunculopontine nucleus; VTA, ventral tegmental area. **b** Gene expression patterns spatially mapped in brain sections using fluorescent in situ hybridization (FISH) (original data reprinted in Fig. 2) allowed the outlining of molecularly profiled domains within mouse STN: STNa (ventromedial STN), STNb (central STN), STNc (dorsal STN), STNds (dorsal strip of STN showing GABA-positive, rather than glutamatergic, mRNA profile). Gene expression (mRNAs) present throughout the STN structure listed in the text box; unique gene expression patterns distinct for each domain listed under the domain name. **c** Selected mRNAs, their distribution and extent of co-localization with STN marker Pitx2, as described in the mouse STN. **b**, **c**: For mRNA full name, see main text. Illustrations based on original publication (Wallén-Mackenzie et al. ^67^; published under Open access).

Based on the complexity of functions mediated by the STN, it has been of particular interest to investigate if this structure is composed of molecularly defined neurons that may allow the dissociation of different behaviors. In the context of revealing transcriptional profiles within a cellular cluster or tissue, single-cell (sc) or single-nuclei (sn) RNA sequencing (RNASeq) technology is of particular importance^112–115^. In a recent study, application of snRNASeq in the Pitx2-Cre transgenic mouse line (described above) to direct selectivity to the STN was implemented. This resulted in the identification of six transcriptional clusters, all positive for *Pitx2*, and represented by strong expression of the glutamatergic marker *Vglut2* (*Slc17a6*) and sparse expression of GABA markers^67^. From the abundance of genes representing different clusters, 16 mRNAs were selected for histological analysis using fluorescent in situ hybridization, FISH, to visualize the mRNA distribution in brain tissue sections covering the entire subthalamic area (included in Table 1, original data re-printed in Fig. 2; illustrated in Fig. 3). Spatial mapping of these 16 transcription products confirmed the distribution of *Vglut2* and *Pitx2* mRNAs throughout the mouse STN, and also identified a similar STN distribution of additional mRNAs. This included the serotonin receptor subtype 2c, *5´Htr2c*, and *Neurexophilin 1* mRNAs. Further, FISH analysis on serial sections allowed the definition of three major STN domains, based on clusters of combinatorial gene expression. For simplicity, these spatio-molecular domains were referred to as STNa, STNb, STNc, with STNa representing the medial-most domain; STNb, the central domain, and STNc, the lateral-most domain (Figs. 2, 3)^67^.

These three STN domains in the mouse were similar, but not identical, to the territories previously described for primate STN in terms of parvalbumin mRNA (for mouse here abbreviated *PV*) and Calbindin 2 (*Calb2*) mRNA such that *PV* mRNA was dominant laterally and decreased in a lateral-medial distribution, while *Calb2* mRNA was most dominant medially. However, in the mouse STN, *Calb2* was primarily detected in a spatially restricted cluster within the medial STNa domain, rather than distributed in a medial-lateral gradient as reported for the primate STN. This is a key difference between mice and primate. Interestingly, another molecular marker was identified in the mouse by snRNASeq which by histological analysis showed a similar distribution pattern as has been described for *Calb2* in primate STN. This was Collagen Type XXIV Alpha 1 Chain (*Col24a1*) mRNA, which was distributed in an opposite gradient compared to *PV* mRNA. The largely opposite expression of *PV* and *Col24a1* mRNAs was critical to the partitioning into three main domains in the mouse STN: STNa (*Col24a1*^*+*^*/PV*^*-*^), medial-most domain; STNb (*Col24a1*^*+*^*/PV*^*+*^), the central domain; STNc (*Col24a1*^*-*^*/PV*^*+*^), the lateral-most domain.

In addition to the three major spatio-molecular domains defined in the mouse STN, one small dorsal strip (termed STNds) was described, which differed in gene expression from the rest of the STN. For example, STNds was positive for Neurexophilin 1 but negative for Neurexophilin 4, while STNa, STNb, and STNc were all positive for both of these. Another distinguishing feature of STNds was positive labelling for Ga*d* mRNA rather than *Vglut2* mRNA, strongly indicating a GABAergic identity^67^. It remains to fully validate if STNds is a bona fide STN structure. One can imagine that stimulating electrodes placed in, or merely affecting, the STNds would give a strikingly rather different result than if they targeted the STNa, STNb, or STNc (Fig. 3B, C).

Further, histological mapping in serial brain sections showed that some markers identified in the snRNASeq experiment were not primarily expressed in the STN but were found to be more useful as markers of anatomically adjacent structures. This included *Tac1* mRNA which was identified as a novel marker for the para-STN, the hypothalamic structure directly adjoined with the medial aspect of the STN (Fig. 3B)^67^.

The transcriptomics-based approach combined with spatial histological mapping in the subthalamic area enabled the identification of a range of molecular markers (gene expression patterns) that allowed dissociation of the STN structure into spatio-molecular defined domains and also the identification of molecularly defined subpopulations in adjacent areas. These anatomically neighbouring areas included the para-STN, and also zona incerta, and the mamillary and retro-mamillary nuclei (these latter structures not further reviewed here)^67^.

Co-expression studies of *Col24a1* and *Calb2* genes would be of interest to examine if they are localized in the same or different neurons within STNa domain. Knock-out of the *Col24a1* gene in mice leads to increased grip strength (The International Mouse Phenotyping Consortium (IMPC)), indicative of motor function and deviating from the tripartite model that the dorsal region regulates motor control. Motor dysfunction in the STN may thus be related to absence Col24a1 function within the STN, of interest to address in future study. Further, it would be of interest if *Col24a1*-positive neurons are more correlated with limbic function of the STN, considering their presence in the ventro-medial tip of the mouse STN, the area which in primates is referred to as the limbic tip based on its projections to limbic brain areas such as VP, as described above. The implementation of the promoters for these spatially restricted expressed genes as drivers of Cre and Flp recombinases in viral-genetics and conditional knockout experiments holds promise for securing a higher level of resolution in studies of the anatomical-functional organization of the STN.

Functional analysis of neuronal STN populations in mice have demonstrated regulation of emotional, locomotor, reward, motivation, and arousal behavior. For example, optogenetic activation of Pitx2^+^ or Gabrr3^+^ neurons of the STN inhibited locomotion and increased repetitive self-grooming behavior in mice^116,117^ (discussed further below). Distinctive STN populations regulating different behaviors have also been demonstrated in human studies. Single neuron recording using micro-electrode recording in the STN of PD patients delineated two spatially distinct STN populations responding to valence and arousal^118^.

In summary, most protein localization studies were conducted in human or monkey brain tissue (Table 1). On the other hand, mRNA studies of the STN were mainly conducted in rodents, but also some in primates (Table 1). The major distinguishing markers of dorsal and ventral STN are Parvalbumin and Calbindin 2, respectively, that show similar (but not identical) distribution in human and rodent tissue. In addition, *Col24a1* mRNA was recently identified in mouse STN as a marker for the medio-ventral and central parts of the STN.

Early studies in the 1900’s disputed whether the STN was a homogeneous structure with no subdivision or two subdivisions^119,120^, followed by the proposal of three subdivisions forming the tripartite organization of the STN^42,121–123^. However, a detailed summary of studies reported between 1925 and 2010 presented by Keuken et al., 2012 indicates a more complex organization of the STN^49^. This is supported by recent studies that have extended analysis across the STN revealing three-dimensional (3D) immunoreactivity of a number of molecular markers within the STN and showing gradient distribution patterns across rostral to caudal and dorsal to ventral plane within the STN in postmortem human brain^33,34^. The localization of gene expression and final protein products as outlined above have expanded the early proposals of cytoarchitecture to reveal a highly complex organization of the STN.

Gene expression studies in rodent studies reveal similar but not identical expression patterns to monkey and humans. The relatively highly conserved expression patterns allow neural modulation in rodents using cutting-edge technologies with high spatial selectivity and precision to then translate back into understanding STN organization in humans.

### STN-regulated behaviors and STN-DBS side-effects

Most evidence of STN functions in behaviors other than motor function emerges indirectly from DBS in PD patients. While improving motor symptoms, some PD patients who underwent STN-DBS have reported weight gain, depression, development of addictive behaviors and other non-motor symptoms^9,23,124–127^. Due to the complex and progressive pathology of PD, along with pre-existing conditions and often in combination with pharmacological treatments, it is difficult to explicitly identify STN contributions in non-motor functions, and to specify if reported adverse effects are due to STN-DBS or due to the progression of the disease and/or pharmacological medications. Direct evidence of STN contributions come from animal models applying STN lesion, DBS, optogenetics and pharmacological manipulations, and that report altered movement, reward, feeding, addiction and impulsive behavior^128–131^. The major functions regulated by the STN are grouped by motor behavior, and associative and limbic functions. These in turn, are dependent on anatomical, molecular, cellular organizations alongside, afferent and efferent projections, forming distinct neurocircuitry.

### Motor function

Activation of the STN is correlated with inhibition of movement via the indirect and hyperdirect pathways, both of which the STN is a critical component^132,133^. Studies of humans show that dorsolateral STN (dlSTN) mainly regulates motor functions, electrode placement for DBS in PD cases is targeted to the dorsolateral STN^134,135^. Pharmacological GABAergic inhibition of the STN in non-human primate model of PD further revealed mediolateral regionalisation within dlSTN where the medial region of dlSTN regulates upper limb and lateral dlSTN is associated to lower limbs^136^. Anterograde tracing studies demonstrated topographically organized cortical afferents to dlSTN, forming the hyperdirect pathway^46^.

The STN is part of the hyperdirect and indirect pathway of the basal ganglia motor circuitry. The indirect pathway works antagonistically to the direct pathway where the direct pathway promotes movement and the indirect pathway inhibits movement, exerting balanced control over movement^137^. Different populations of striatal neurons within the basal ganglia give rise to the direct and indirect pathways. Optogenetic activation under the control of DR1 or DR2 promoters leads to opposing results on movement. Activation of the direct pathway (DR1) increases movement whereas activation of indirect pathway (DR2) decreases movement, respectively^133^. The STN mainly receives inhibitory projections from globus pallidus (GP) *externa* (GPe) and extends glutamatergic projections to substantia nigra *pars reticulata* (SNr) and GPi^138,139^. In line with the relay of the indirect pathway, optogenetic inhibition of STN neurons promotes movement^117,131,140,141^ while optogenetic excitation of the STN inhibits movement, reducing specific locomotion tasks^117,131^. As described above, similar results have been observed upon conditional reduction of *Vglut2* gene expression levels in STN in mice which induces hyperlocomotion without impacting on limbic and cognitive functions^106,108^. Curiously, optogenetic excitation of the STN did not only reduce motor tasks. Instead, some behaviors were increased, such as self-grooming and jumping behaviors which were initiated immediately upon photo-stimulation of the STN^117^. Together, these results demonstrate that STN neuronal activity is capable of bidirectional motor regulation.

### Limbic function

The STN is an important mediator of reward-seeking behavior^142–144^. Significant weight gain has been reported by a number of PD patients following STN-DBS^124^. Potential mechanisms for shift in weight from clinical studies include decrease in energy expenditure^127^, changes in neuropeptide level (neuropeptide Y and ghrelin)^145^ and increase in food intake^146^. STN lesions in rodents show increase in motivation for food^147,148^. Real-time calcium imaging using fibre photometry in mice evoked enhanced activity of STN neurons during feeding behavior^130^. Increase in STN neural firing activity was specific for sweet food and standard rodent chow but not for bitter taste. The extent of neural activity was dependent on the valence and palatability of food. Bidirectional optogenetic manipulation during feeding behavior showed that inhibition of the STN led to increased food intake while activation of the STN decreased food intake, but only during the dark phase (period when nocturnal animals consume food)^130^.

Limbic functions with the STN are most likely influenced by input from the VP^149^. Neural tracer studies in monkeys reveal that the VP projects selectively to the ventral region of the STN, correlating to the limbic domain^46,47^. Similar connectivity is found in rats, where the dorsal lateral VP projects to the dorsal medial STN^71,150–153^. In a rodent model of alcohol addiction, the pathway from VP to the STN is active during relapse to alcohol-seeking, and inhibition of this pathway reduced relapse to alcohol-seeking without affecting locomotion^149^.

STN is involved in the motivation to respond for a variety of drugs of abuse including methamphetamine and cocaine^18,142,148,154,155^. STN-DBS decreased the effort for cocaine intake but increased food intake, demonstrating differential patterns of activity in the STN for drugs of abuse and natural rewards^148^. Low-frequency neuronal oscillations of theta and beta bands in the STN corelated with increased escalation of cocaine intake^18^. Moreover, DBS at low frequency of 30 Hz reduced compulsive cocaine-seeking in rats^19^. Administration of the neuropeptide oxytocin reduces relapse to Methamphetamine-seeking behavior^156^.

A recent study implemented Cre-driven optogenetics using Pitx2-Cre, Vglut2-Cre and PV-Cre to address the role of STN in emotional affect. It was shown that excitation of the whole STN using either Pitx2-Cre or Vglut2-Cre resulted in behavioral avoidance and cue-induced aversion. In contrast, PV-Cre, implemented to tease out the dorsal STN, did not give as the same potent aversive response. This result is a strong indicator that the PV-positive subpopulation of STN is not a primary driver of aversion mediated by the STN, but that STN aversion neurons remain to identify. The study also found an indirect pathway to the lateral habenula, a key hub for aversion and depression, via STN-projections to VP and GPi (called entopeduncular nucleus in rodents). In addition, this study demonstrated striking similarity in Pitx2, Vglut2, PV and Tac1 expression patterns between mouse and macaque monkey and identified a clear boundary between STN and para-STN defined by a mutually exclusive expression of Pitx2 (STN) and Tac1 (para-STN). Excitation of STN caused aversive learning, while similar excitation of para-STN did not results in a conditioned response. This study shows that the implementation of spatially selective promoter activities holds promise towards anatomical-functional decoding of the STN^157^.

Overall, changes in STN neural oscillation, activity and connectivity from the VP and neuropeptides such as neuropeptide oxytocin have been demonstrated to regulate limbic functions impacted in feeding and substance use disorders. Together, these findings provide insight towards refined approaches to target limbic dysfunctions. STN has been identified as a target for DBS treatment of addiction^158,159^. Further characterization of subpopulations of STN neurons engaged in specific limbic functions will be important.

### Cognitive function

STN contributions in cognition have been demonstrated in action control, decision-making, attention, executive functions and verbal learning and memory^160,161^. The STN is strongly associated with cognitive behavior in monkey and humans, specifically in action selection between correct and incorrect behaviors^162–165^. Cortical afferents to the ventromedial STN region, the associative area of the STN in accordance with the tripartite model, have been characterized in primates and rats^166–168^. STN-DBS studies in clinical PD have identified a decline in cognitive activity in working memory, response inhibition and verbal functions when comparing these abilities pre-operative and post-operative DBS^169–173^. In OCD cases, STN-DBS impacts on decision-making and response inhibition^163,174^. Animal models with STN lesions show impaired response inhibition^175,176^ and increase in impulsivity^177^. Optogenetic inhibition of medial prefrontal control projections to STN impaired spatial working memory but not anxiety^178^. Changes in THETA oscillations in the STN regulates response inhibition^179,180^. Cognitive functions in the STN are dependent on oscillatory dynamics in the STN and inputs from the cortex^178,181^.

Recent studies clearly demonstrate the capacity of STN to regulate motor control emphasizing its essential role of the STN in motor regulation. Yet the specific role of STN in motivational and cognition requires further studies. In motivational behavior, the changes in STN neural oscillations and connectivity to the VP are established. However, the contributions of dopamine inputs in STN in motivational behavior is an important area that remains to be investigated, especially in the realm of motivational dysfunction in PD patients.

### STN-related neurological disorders

The STN has been implicated in several neurological disorders, including PD, progressive supranuclear palsy (PSP), argyrophilic grain disease (AGD), Huntington’s disease, spinocerebellar ataxia 3 and 7, and dentatorubral-pallidoluysian atrophy (DRPLA)^36,182,183^. Apart from PD, in which no degeneration of STN neurons have been identified, the pathology of all other STN disorders includes neuronal loss and astrocytic gliosis in the STN^51^. In the case of spinocerebellar ataxia type 3, brownish discoloration of the STN is also a feature^32,184–186^. In Huntington’s chorea disease, the STN undergoes 25% reduction in volume and number of STN neurons^183^. PSP is a degenerative disorder that effects the STN and is often misdiagnosed as PD due to the PSP and PD diseases displaying similar motor symptoms^182^. While the STN of PSP patients undergoes neurodegeneration with significant loss of neurons, neurofibrillary tangles and gliosis, the STN of PD patients remain anatomically intact^36,184^.

PD is the second most common neurodegenerative disorder^187^. Lesion of the STN has been used as a treatment for PD in the past, providing therapeutic effects on motor symptoms^188,189^. STN-DBS provides a safer and more effective alternative, as it is a reversible and adjustable treatment. Despite the pivotal role of the STN in PD symptoms and treatment, our knowledge of its pathology during PD is limited. It has been established that the STN of PD patients is hyperactive with continuous abnormal ‘bursting’ which fires at a higher rate^42,190^. This hyperactivity not only contributes to the motor symptoms experienced in PD but shows correlation to the severity of motor deficits as well^191,192^. In PD, a loss of dopamine-producing neurons of the SNc results in decreased dopaminergic activity in the basal ganglia, causing dysfunction of the basal ganglia pathways. It is hypothesized that this results in excessive activity of the STN causing abnormal activation of the indirect pathway. This increased STN activity increases involuntary movements and decreased the ability to execute intended movement, characterized by the motor symptoms seen in PD patients^191^.

### Cellular changes in STN-related disorders

The STN is a major treatment target for advanced stage PD, treatment resistant OCD, essential tremor and dystonia^8,123,193–197^. Efficacy and tolerability of DBS in Tourette’s syndrome is currently in trials for treatment of severely affected and otherwise treatment-resistant patients. However, there are no direct links to cellular or anatomical pathology of the STN in either disorder. Instead, interventions aim at targeting altered firing activity of STN neurons. In PD patients, the dysregulation in STN neural firing is correlated to the motor deficit severity, rather than changes in STN anatomy^198^.

Alterations in STN neurotransmission were proposed to be driven by GABA inputs from the GP and dopamine inputs from the SNc^199^. Post-mortem analysis has shown decreased density of GABA_B_ receptors in the caudate putamen and GPe in PD patients compared to controls^200^. GABA_A_ receptors mediate fast and GABA_B_ mediate slow inhibitory responses in the STN. GABA_A_ and GABA_B_ receptor subunits are differentially localized in the human STN where GABA_A_ was identified as more intensely distributed in the ventromedial region of the STN and GABA_B_ subunits were detected throughout the STN^79^. Further analysis of GABA receptor subunits in the STN of PD cases should be an area of further investigation.

Among molecular markers in the STN reported in rodent, monkeys and humans (listed in Table 1), very few have been examined in STN-related disorders. However, it has been shown that the segmentation of PARV and CALB2 immunoreactivity in control post-mortem human brain tissues and PD cases were unchanged^36^, leaving the idea that the STN is anatomically intact in PD cases. One study has reported the presence of Lewy bodies in the STN of PD cases^201^. This finding suggests that instead of cell loss, there may be cellular dysfunction of STN neurons in PD.

The cellular pathologies identified within the STN tend to be disease-specific with either cell loss, aberrant neural firing pattens or changes at the protein level. Only a handful of cellular markers have been examined in STN. However, recent expression studies in healthy human STN have identified spatial expression of a range of proteins in the STN to the examined in disease cases.

### Future directions

The molecular organization of the STN is strikingly heterogeneous. This heterogeneity, of which more is likely yet to be revealed, provides an increased understanding towards the neurobiological underpinnings of the roles played by STN in motor, limbic and cognitive regulation. While we are likely only at the doorstep of understanding the complexity of this clinically crucial brain area, the association of STN heterogeneity with the regulation of distinct behaviors will be significant throughout the fields of physiology, motivation, movement, cognition, psychology and more. For example, future therapeutic approaches such as optogenetic stimulation which target specific cell types or hybrid application of electric and optogenetic stimulation can refine current DBS approaches to reduce adverse or off-target effects^202^.

This review focused on summarizing current knowledge of long-established and newly described molecular and cellular markers within the STN. Future directions include firstly to functionally characterize such markers in the context of the different roles played by the STN, and secondly to assess their putative regulation in STN-related diseases. Further studies are required to extend current knowledge of the internal architecture of the STN and its plethora of roles in neurocircuitry and behavioral control. The examination of molecular profiles within the STN in normal brain physiology and in neurological disorders will aid towards a better understanding of STN´s role in health and disease, and add information towards more precise brain maps, which in turn can help towards refined treatment approaches.
